# Hydrogen Sulfide Preconditioning Protects Rat Liver against Ischemia/Reperfusion Injury by Activating Akt-GSK-3β Signaling and Inhibiting Mitochondrial Permeability Transition

**DOI:** 10.1371/journal.pone.0074422

**Published:** 2013-09-13

**Authors:** Qingqing Zhang, Hailong Fu, Hao Zhang, Fengying Xu, Zui Zou, Meng Liu, Quanxing Wang, Mingyong Miao, Xueyin Shi

**Affiliations:** 1 Department of Anesthesiology, Changzheng Hospital, Second Military Medical University, Shanghai, China; 2 National Key Laboratory of Medical Immunology and Department of Immunology, Second Military Medical University, Shanghai, China; 3 Department of Biochemistry and Molecular Biology, Second Military Medical University, Shanghai, China; University College London, United Kingdom

## Abstract

Hydrogen sulfide (H_2_S) is the third most common endogenously produced gaseous signaling molecule, but its impact on hepatic ischemia/reperfusion (I/R) injury, especially on mitochondrial function, remains unclear. In this study, rats were randomized into Sham, I/R, ischemia preconditioning (IPC) or sodium hydrosulfide (NaHS, an H_2_S donor) preconditioning groups. To establish a model of segmental (70%) warm hepatic ischemia, the hepatic artery, left portal vein and median liver lobes were occluded for 60 min and then unclamped to allow reperfusion. Preconditioning with 12.5, 25 or 50 μmol/kg NaHS prior to the I/R insult significantly increased serum H_2_S levels, and, similar to IPC, NaHS preconditioning decreased alanine aminotransferase (ALT) and aspartate aminotransferase (AST) levels in the plasma and prevented hepatocytes from undergoing I/R-induced necrosis. Moreover, a sub-toxic dose of NaHS (25 μmol/kg) did not disrupt the systemic hemodynamics but dramatically inhibited mitochondrial permeability transition pore (MPTP) opening and thus prevented mitochondrial-related cell death and apoptosis. Mechanistic studies revealed that NaHS preconditioning markedly increased the expression of phosphorylated protein kinase B (p-Akt), phosphorylated glycogen synthase kinase-3 beta (p-GSK-3β) and B-cell lymphoma-2 (Bcl-2) and decreased the release of mitochondrial cytochrome c and cleaved caspase-3/9 levels. Therefore, NaHS administration prior to hepatic I/R ameliorates mitochondrial and hepatocellular damage through the inhibition of MPTP opening and the activation of Akt-GSK-3β signaling. Furthermore, this study provides experimental evidence for the clinical use of H_2_S to reduce liver damage after perioperative I/R injury.

## Introduction

Hepatic ischemia/reperfusion (I/R) injury influences the prognosis of patients in a variety of clinical contexts, including transplantation, liver resection surgery, trauma and hemorrhagic shock [1,2]. However, the current therapeutic treatment strategies used to prevent hepatic I/R injury are not optimal because the underlying molecular mechanisms remain unclear. Evidence suggests that liver I/R injury occurs along with an inflammatory process that causes cellular damage due to complex factors, such as the production of reactive oxygen species (ROS), chemokines, and cytokines [3]. The disruption of intracellular energy metabolism, which results in adenosine triphosphate (ATP) depletion, an accumulation of sodium and edema [4], suggests that mitochondria play an important role in I/R injury.

Mitochondrial permeability transition pore (MPTP) opening in the inner mitochondrial membrane has been implicated in I/R injury. It causes a disruption of the proton gradient and electrical potential across the inner mitochondrial membrane, which leads to an influx of solutes and water and eventual rupture of the outer membrane, culminating in necrotic cell death. In addition, cytochrome c, apoptosis-inducing factor (AIF) and Ca^2+^, which are released from the mitochondria, activate procaspase-9 and other members of the caspase family [5,6,7,8], which lead to apoptosis. Previous studies have shown that inhibiting MPTP opening by activating intracellular signal transduction pathways, such as the phosphoinositide 3'-OH kinase/protein kinase B (PI3K/Akt), extracellular regulated protein kinases (ERK1/2) and the Janus kinase/signal transducer and activator of transcription (JAK/STAT) pathways, can alleviate I/R injury [9,10,11,12].

For many years, hydrogen sulfide (H_2_S) was considered a toxic agent that, at high concentrations, could reversibly inhibit complex IV (cytochrome c oxidase), the terminal enzyme complex in the electron transport chain [13]. Recently, H_2_S has been recognized as a third inorganic gaseous mediator [14,15,16], in addition to nitric oxide (NO) and carbon monoxide (CO), and can thus influence various cellular processes. H_2_S is produced by cystathionine-β-synthase, cystathionine-γ-lyase and 3-mercapto-pyruvate-sulfur-transferase in mammalian cells [17]. Two-thirds of H_2_S molecules dissociate into hydrogen ions (H^+^) and bisulfide ions (HS^-^) under physiological conditions [18]. Therefore, sodium hydrosulfide (NaHS) can be administered as a water-soluble H_2_S donor. The diverse physiological functions of H_2_S make it capable of protecting the heart [19], brain [20], liver [21,22,23], kidney [24], and lung [25] against I/R injury when given at sub-toxic doses. In the liver, the underlying mechanisms of protection appear to include suppressing oxidative stress via antioxidant activities, reducing inflammatory mediators, such as tumor necrosis factor-α (TNF-α), interleukin-10 (IL-10) and intercellular cell adhesion molecule-1 (ICAM-1), and reducing hepatocyte apoptosis. Additionally, H_2_S can up-regulate B-cell lymphoma-2 (Bcl-2) expression [22,23]. However, whether H_2_S preserves mitochondrial function in hepatic I/R injury remains unclear. Therefore, we employed a rat model of 70% warm hepatic I/R to elucidate the role of H_2_S preconditioning on the susceptibility of the MPTP and the underlying mechanism of H_2_S-mediated protection of the liver.

## Materials and Methods

### Materials

NaHS was purchased from Sigma Chemical Co. (Sigma, St. Louis, MO). Antibodies for Akt, phosphorylated Akt (p-Akt), GSK3β, phosphorylated GSK-3β (p-GSK-3β), Bcl-2, activated caspase-3/9 and cytochrome c were purchased from Cell Signaling Technology (CST, Boston, MA). The Calcium Green-5N probe was purchased from Invitrogen (Carlsbad, CA, USA). All other chemical reagents were of pure analytic grade.

### Animals and surgery

**Figure 1:**
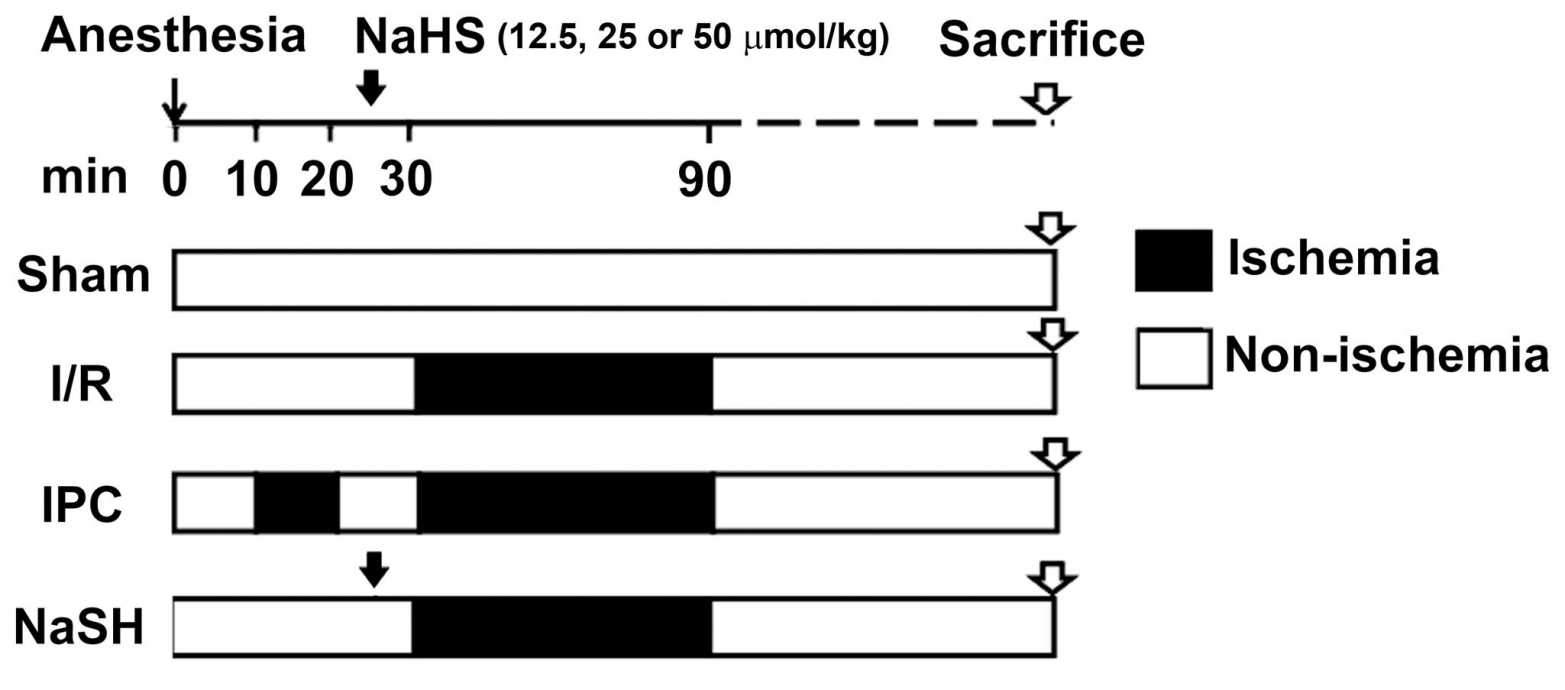
Experimental design. Rats underwent 60 min of ischemia followed by 4 or 24 h of reperfusion. Ischemia preconditioning (IPC) consisted of 10 min of ischemia and 10 min of reperfusion before the full 60 min of ischemia insult. For the NaHS groups, different doses of NaHS (12.5, 25 or 50 μmol/kg) were injected intravenously 5 min prior to the onset of liver ischemia. I/R, ischemia/reperfusion.

Eight-week-old male Sprague–Dawley rats (weighing 200-220 g), obtained from Sino-British Sippr/BK Lab Animal Ltd (Shanghai, China), received a standard laboratory diet containing 12% fat, 28% protein, and 60% carbohydrates and were housed under SPF conditions according to the institutional guidelines. The study protocol was approved by the Committee on the Ethics of Biomedicine Research of the Second Military Medical University (Approval file number: 2009LL029). Our experimental design is illustrated in Figure 1. Briefly, animals were randomly divided into one of the following groups: the sham operation (Sham) group; hepatic ischemia/reperfusion (I/R) group; ischemia preconditioning (IPC) group; and three NaHS preconditioning groups, which received 12.5, 25 or 50 μmol/kg of NaHS prior to the induction of I/R injury. IPC was performed by routine vascular clamping prior to the induction of long-term ischemia. Given that IPC has been shown to have protective effects through the activation of multiple protective signaling pathways during I/R [26,27], we chose it as a positive control. All animals were fasted for 12 hours prior to surgery and anesthetized with pentobarbital (1%, 40 mg/kg) intraperitoneally. The left femoral vein was exposed and cannulated to infuse 0.9% saline and drugs, while the left femoral artery was cannulated to measure the heart rate (HR) and the mean arterial pressure (MAP). A model of segmental (70%) warm hepatic I/R was established as described previously, with minor modifications [28]. Briefly, after a midline laparotomy, an atraumatic clip was used to interrupt the arterial and portal venous blood supply to the left and median liver lobes for 60 min. Reperfusion was initiated by removal of the clamp. Sham-operated rats underwent the same procedure but without vascular occlusion. IPC rats received 10 min of ischemia and 10 min of reperfusion before the 60 min ischemic insult. Rats in the NaHS group were treated with different NaHS solutions (12.5, 25 or 50 μmol/kg) through the left femoral vein 5 min prior to the onset of liver ischemia, and the other groups received a comparable volume of saline. During the surgery, the animals’ core body temperatures were maintained at 37°C using heat pads and lamps. The rats were sacrificed after 4 or 24 h of reperfusion, and liver and serum samples were collected for analysis.

### Measurement of H_2_S concentration and aminotransferase levels in the plasma

The rats were euthanized 4 h after reperfusion. Blood samples were immediately collected from the heart and centrifuged to obtain plasma. H_2_S concentrations in the plasma were measured as previously described [21]. Briefly, 75 μL of plasma was mixed with 250 μL of 10% trichloroacetic acid, 250 μL of 1% zinc acetate and 150 μL of distilled water in a 1.5 ml Eppendorf tube. Subsequently, 133 μL of 20 mmol/L N-dimethyl-p-phenylenediamine sulfate and 133 μL of 30 mmol/L FeCl_3_ were added to the plasma, and the reaction mixture was incubated at room temperature (25°C) for 10 min followed by centrifugation at 32,900×g for 5 min. The absorbances of the resulting supernatants were read at 670 nm with a micro-plate reader (Model 680, BioRad, USA). All samples were assayed in duplicate, and the concentrations of each sample were calculated based on a standard curve constructed with known concentrations of NaHS. The serum levels of alanine aminotransferase (ALT) and aspartate aminotransferase (AST) were determined with a multi-analyzer (H-7600; Hitachi Ltd., Tokyo, Japan).

### Histology

Liver tissue samples were collected, ﬁxed with formalin and embedded in paraffin for histological analyses. Liver sections (4 μm) were stained with hematoxylin-eosin (H-E) and analyzed in a blinded manner. The severity of I/R injury was graded with Suzuki’s criteria [29], with some modifications. Briefly, in this classification, sinusoidal congestion, hepatocyte necrosis, and ballooning degeneration were graded from 0 to 4. Samples without any necrosis, congestion or centrilobular ballooning were given a score of 0, whereas samples with severe congestion, ballooning degeneration and greater than 60% lobular necrosis were given a score of 4. A terminal deoxynucleotidyl transferase-mediated dUTP nick-end labeling (TUNEL) stain was performed with a commercial kit from Roche (Rotkreuz, Switzerland), according to the manufacturer’s instructions. In each section, areas without significant necrosis in 10 different visual fields (400×) were analyzed for TUNEL-positive cells. A TUNEL index was calculated by counting the total nuclei and the cells with brown nuclei in the peri-infarcted area of five visual fields. The TUNEL index was determined using the following formula: (number of stained cells/number of stained cells + number of unstained cells) *100. Four sections of tissues were analyzed for each group.

### Systemic hemodynamic status measurement

To determine the systemic hemodynamic status of the animals, the rats were first anesthetized with pentobarbital. A polyethylene catheter (PE 50; Becton Dickinson, Sparks, MA) was advanced through the left femoral artery and into the descending aorta to measure the central mean arterial pressure (MAP). The catheters were flushed intermittently with saline solution containing 2.5 IU/mL bovine heparin. The MAPs and heart rates (HRs) of the rats were measured with a multi-channel physical recorder (MPA 2000, Alcott Biotech, Shanghai, China) during the I/R insult.

### Mitochondria isolation

Mitochondria were isolated by gradient centrifugation as we previously described [25]. Briefly, fresh liver tissues (1 g) were homogenized with 8 ml of isolation buffer containing 220 mmol/L D-mannitol, 70 mmol/L sucrose, 10 mmol/L Tris-HCl, 1 mmol/L EGTA, and 0.4% bovine serum albumin (pH 7.4). The homogenates were centrifuged at 850×g for 10 min to collect supernatants, followed by centrifugation at 10,000×g for an additional 10 min. The mitochondrial pellet was resuspended in a final wash buffer containing 220 mmol/L D-mannitol, 70 mmol/L sucrose, and 10 mmol/L Tris-HCl (pH 7.4). The total protein concentration was determined with the biuret method and was calibrated to a standard curve generated with bovine serum albumin.

### Calcium retention capacity

A calcium retention capacity (CRC) assay was adapted from a previously described method [25]. Briefly, the CRC was defined as the amount of Ca^2+^ required to trigger a massive Ca^2+^ release in isolated liver mitochondria. It was used as an indicator of the resistance of the MPTP to opening after matrix Ca^2+^ accumulation and is expressed as nmol CaCl_2_ per mg mitochondrial protein. The extramitochondrial Ca^2+^ concentration was determined with a fluorescence microplate reader controlled by SOFTmax PR software (Molecular Devices, Sunnyvale, CA, USA) in the presence of 1 µmol/L Calcium Green-5N molecular probe, with the excitation and emission wavelengths set at 505 and 535 nm, respectively. The fluorescence scan interval was set at 12 s. Isolated mitochondria (2 mg total protein) were suspended in 1 ml of incubation buffer (220 mmol/L D-mannitol, 70 mmol/L sucrose, 1 mmol/L Pi-Tris, 10 mmol/L Tris-MOPS, 5 mmol/L glutamate-Tris, and 2.5 mmol/L malate-Tris, pH 7.4, containing 0.01% [w/v] bovine serum albumin and 1 μmol/L of the Ca^2+^ indicator Calcium Green-5N) in a clear 24-well plate. After a 120 s pre-incubation period, 10 nmol CaCl_2_ pulses were performed every 60 s to calculate the CRC. After sufficient calcium loading, the extra-mitochondrial calcium concentration abruptly increased, indicating a massive release of calcium by the mitochondria as a result of MPTP opening.

### Western blot analysis

The levels of Akt, GSK3β, Bcl-2, caspase-3 and caspase-9 were determined in liver lysates. Cytochrome c levels were determined in cytoplasmic extracts according to the method of Ludovic Gomez [30]. Briefly, liver tissues were homogenized in lysis buffer (Promega, Madison, WI, USA). After removing the nuclei and cell debris by centrifugation at 850×g for 10 min at 4°C, the supernatants were further centrifuged at 10,000×g for 10 min at 4°C. Then, the supernatants were collected for cytoplasmic cytochrome c analysis. The protein concentration of the extracts was determined by the BCA protein assay (Pierce, Rockford, IL, USA). An equal amount of protein from each sample was separated on an SDS polyacrylamide gel and transferred onto a nitrocellulose membrane (Millipore, Temecula, CA, USA). After incubation with the indicated primary antibodies, the blots were probed with a goat anti-rabbit or an anti-mouse secondary horseradish peroxidase (HRP)-conjugated antibody (Santa Cruz, CA, USA) and developed with enhanced chemiluminescence reagents (Pierce). The relative amount of the target protein was normalized to β-actin and analyzed with a Gel Pro Analyzer (Media Cybernetics, Silver Spring, MD, USA).

### Statistical analysis

The hemodynamic data are presented as the median (range). Data within groups were analyzed with a Friedman repeated-measures ANOVA on ranks and a subsequent post-hoc multiple comparison procedure (Dunn method). Differences between treatment groups within one measurement point were analyzed with the Mann-Whitney U rank sum test for unpaired samples. Other data are expressed as the mean ± standard deviation (SD). Statistical analysis was performed with a one-way analysis of variance (ANOVA), and comparisons between tested groups were conducted with LSD tests. SPSS 10.0 (SPSS Inc, Chicago, IL, USA) was used for the statistical analysis. In all cases, a *P* value <0.05 was considered to be statistically significant.

## Results

### H_2_S preconditioning reduces I/R-induced hepatic injury

**Figure 2:**
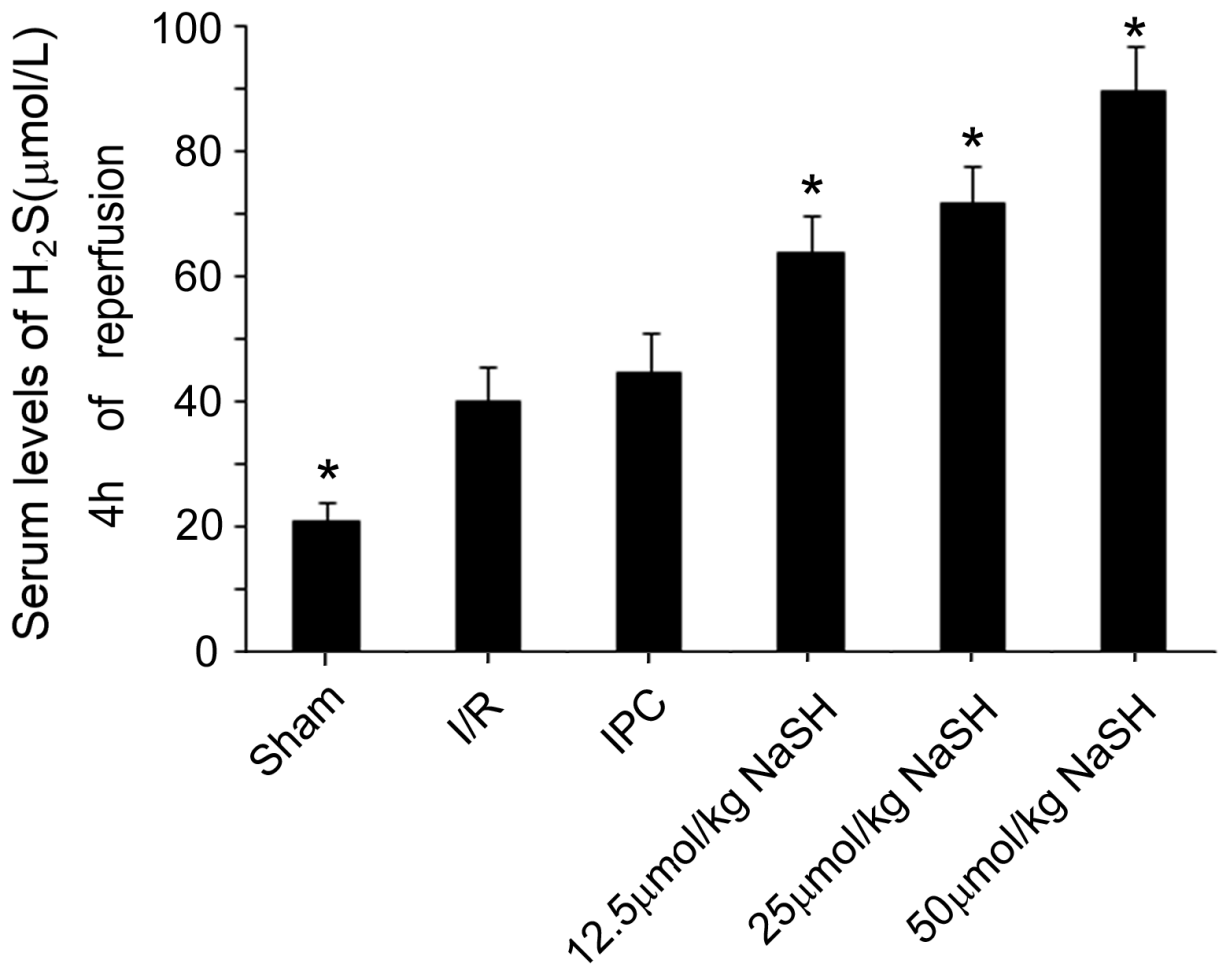
Serum levels of H_2_S. Rats in the different groups were treated as described in Figure 1. Serum levels of H_2_S were assayed in the animals after 4 h of reperfusion. Rats that received a preconditioning dose of 12.5, 25 or 50 μmol/kg NaHS displayed significantly increased serum levels of H_2_S compared to rats in the I/R group. At least six rats were included in each study group. The results are expressed as the mean ± SD. * *P* < 0.05 versus I/R.

**Figure 3:**
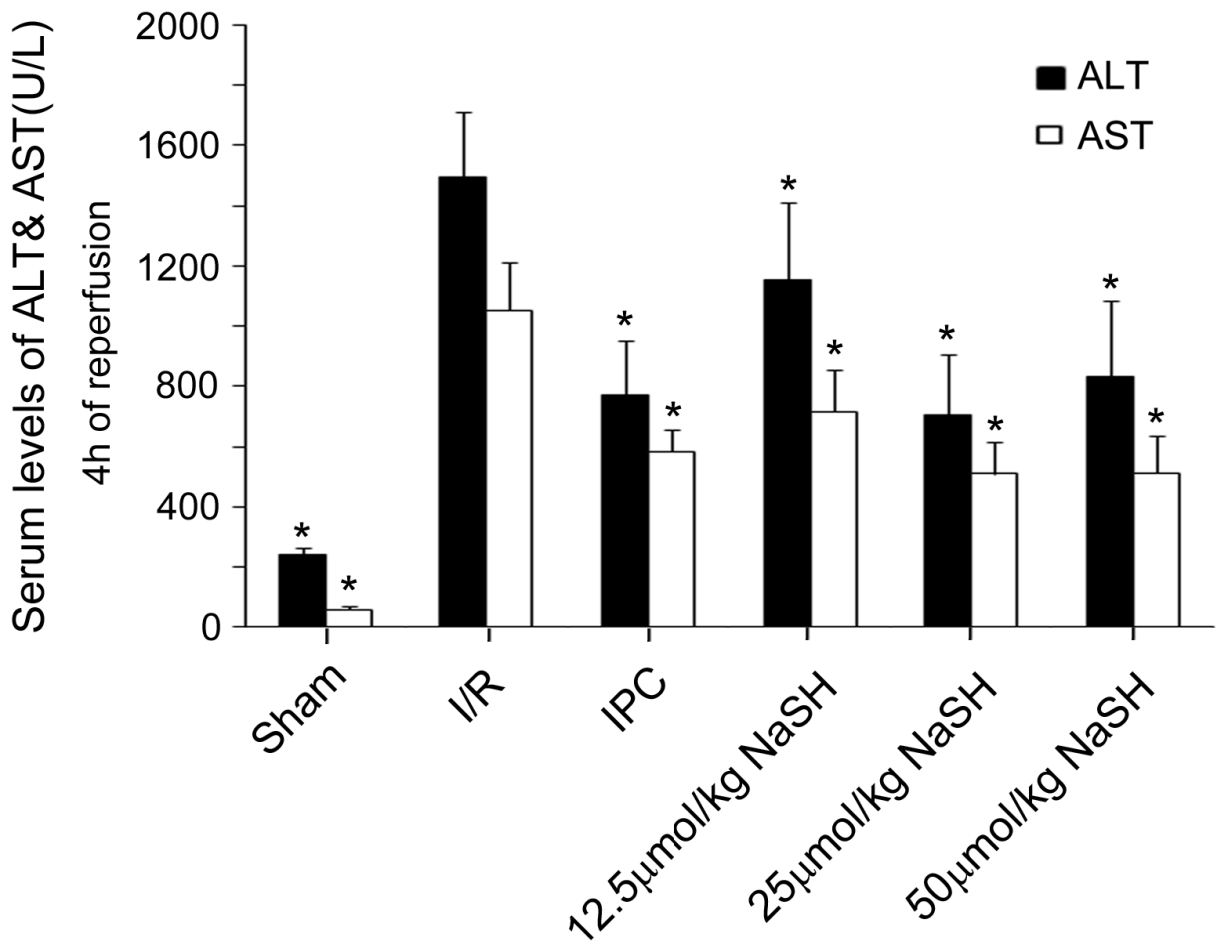
Serum levels of aminotransferase. Rats in the different groups were treated as described in Figure 1. Serum levels for (A) alanine aminotransferase (sALT) and (B) aspartate aminotransferase (sAST) were determined in animals after 4 h of reperfusion. At least six rats were included in each study group. The results are expressed as the mean ± SD. * *P* < 0.05 versus I/R in the same strain.

**Figure 4:**
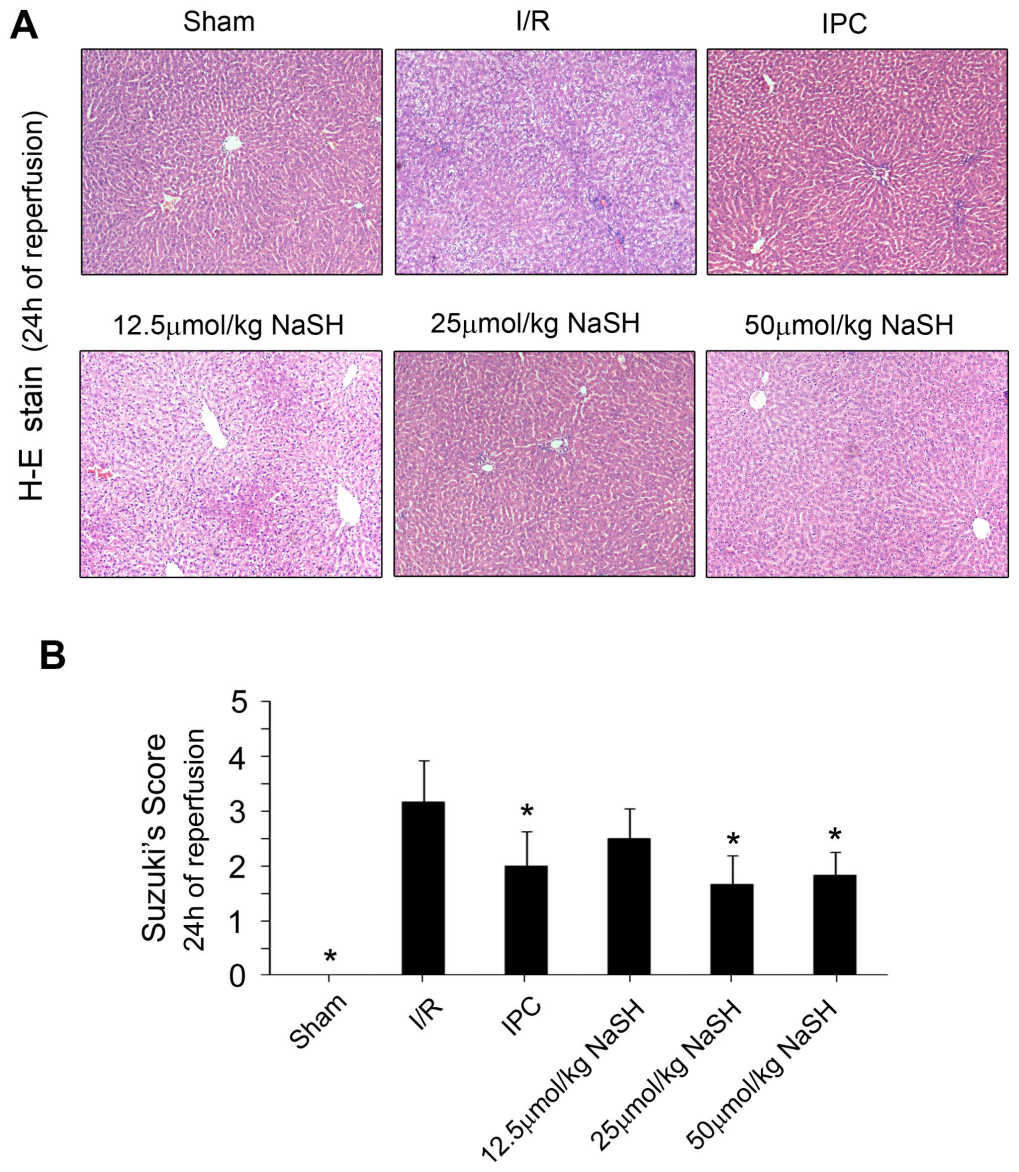
The effects of NaHS preconditioning on liver damage. Rats in the different groups were treated as described in Figure 1. (A) H&E staining of livers collected 24 h after reperfusion (100× magnification). (B) Bar graphs showing the Suzuki’s scores for the tissues. At least six rats were included in each study group. The results are expressed as the mean ± SD. * *P* < 0.05 versus I/R.

To identify the effect of different preconditioning doses of NaHS on hepatic injury, the serum levels of H_2_S, ALT and AST were measured 4 h after reperfusion. Preconditioning with 12.5, 25 or 50 μmol/kg NaHS five minutes prior to the ischemic insult markedly increased the serum concentration of H_2_S (Figure 2) and reduced the serum levels of ALT and AST to varying degrees (Figure 3) compared with the I/R group. These results imply that the NaHS preconditioning alleviated the hepatic damage caused by the I/R injury. However, the reduction of ALT and AST serum levels did not occur in an H_2_S concentration-dependent manner, as no difference could be found between the 25 μmol/kg and 50 μmol/kg NaHS-treated groups (ALT: 706U/L in 25 μmol/kg versus 832 U/L in 50 μmol/kg NaHS; AST: 509 U/L in 25 μmol/kg NaHS versus 512 U/L in 50 μmol/kg NaHS*, P* >0.05). Next, H-E staining was performed on the liver tissues after 24 h of reperfusion, and a Suzuki’s score was calculated to measure the severity of hepatocyte injury. These scores further confirmed the above results. Rats that were preconditioned with 25 or 50 μmol/kg NaHS and rats that received IPC displayed less liver damage (Figure 4A) and lower Suzuki’s scores (Figure 4B) than rats in the I/R group. These results suggested that NaHS preconditioning protects rats from I/R-induced hepatic injury by inhibiting cell death, similar to IPC. However, in the 50 μmol/kg NaHS group, 33.3% of rats presented with dyspnea and died during the surgical procedure, which was likely caused by H_2_S-related lung injury, as previously reported [31]. Therefore, we decided to use a sub-toxic dose of NaHS (25 μmol/kg) to further investigate the protective mechanisms of H_2_S on hepatic I/R.

### H_2_S preconditioning has no effect on systemic hemodynamics during I/R injury

Alterations in systemic hemodynamics and organ blood supply may contribute to I/R injury. Thus, the systemic hemodynamics of rats in the I/R, IPC and NaHS (25 μmol/kg) groups were measured at six different time points (before ischemia; 20, 40 and 60 min after ischemia; and 2 h and 4 h after reperfusion). There was a transient drop in blood pressure during the rapid intravenous injection of 25 μmol/kg NaHS, which was quickly restored to the baseline level after the injection (data not shown). At each time point, the HRs and MAPs were not significantly different between rats in the NaHS group and the I/R and IPC groups (Table 1). These results indicated that NaHS preconditioning did not have an effect on systemic hemodynamics; however, there may be other mechanisms by which H_2_S reduced cell death and protected the liver from I/R injury.

**Table 1:** Parameters of systemic hemodynamic status of the rats.

		**before ischemia Baseline**	**Ischemia**			**Reperfusion**	
			**20min**	**40min**	**60min**	**2h**	**4h**
**Heart rate (Beats·min^-^1**)	**I/R**	**294(279-319)**	**309(298-345)**	**304(292-325)**	**316(302-335)**	**319(298-331)**	**310(278-335)**
	**IPC**	**301(288-317)**	**311(302-331)**	**310(298-335)**	**299(287-315)**	**309(283-335)**	**312(298-325)**
	**NaHS**	**303(281-312)**	**317(298-343)**	**303(288-315)**	**301(278-315)**	**305(289-326)**	**310(292-330)**
**Mean arterial pressure (mmHg**)	**I/R**	**127(119-135)**	**110(90-126)**	**116(90-130)**	**109(91-124)**	**112(98-138)**	**121(91-140)**
	**IPC**	**119(110-138)**	109(98-128)	**110(89-133)**	**114(92-131)**	**120(87-143)**	**122(102-143)**
	**NaHS**	**120(102-130)**	**116(87-130)**	**109(92-129)**	**115(88-136)**	**121(88-135)**	**112(96-139)**

Mean arterial pressure refers to the pressure measured via a polyethylene catheter through the left femoral artery and into the descending aorta (MAP, see the Materials and Methods section). The systemic hemodynamic status before ischemia was set as the baseline. All data are presented as the median (range), and at least eight rats were included in each study group. No significant difference was found in rats in the 25 μmol/kg NaHS preconditioning group compared with rats in the I/R or IPC groups at each time point.

### H_2_S regulates MPTP opening

**Figure 5:**
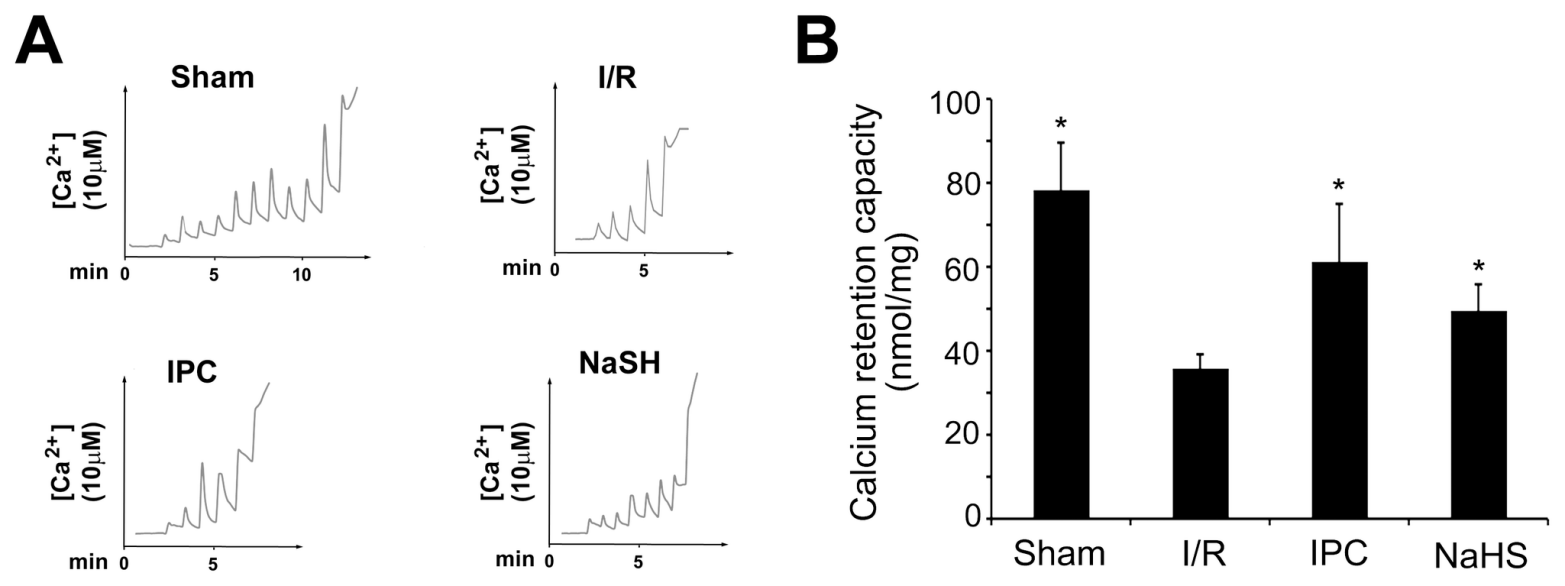
The effects of preconditioning with 25 μmol/L NaHS on mitochondrial calcium tolerance. Mitochondria were isolated from animals from each group that were euthanized after 60 min of hepatic ischemia plus 24 h of reperfusion. Calcium pulses were fluorometrically monitored using the probe Ca^2+^ Green-5N. (A) Determination of extra-mitochondrial Ca^2+^ after subsequent addition of 10 μmol/L CaCl_2_ pulses to mitochondria isolated after 24 h of reperfusion. At the end of the preincubation period, 10 nmol CaCl_2_ pulses were performed every 60 s in 1 ml of 2 mg/ml mitochondria incubation buffer. After sufficient calcium loading, the extra-mitochondrial calcium concentration abruptly increased, indicating a massive release of calcium by mitochondria as a result of MPTP opening. The CRC was then calculated. NaHS preconditioning significantly restored the ability of mitochondria to tolerate calcium induction compared with mitochondria from rats that only received I/R. (B) Calcium retention capacity after 24 h of reperfusion in each group. At least six rats were included in each study group. The results are expressed as the mean ± SD. * *P* < 0.05 versus CRC in the I/R group.

The MPTP is an important master regulator of cell death in I/R injury. Several signaling pathways, such as the PI3K-Akt pathway, Erk1/2 pro-survival kinase pathway and JAK-STAT pathway, regulate the MPTP during reperfusion [11,32]. However, the effects of H_2_S on the MPTP in hepatic I/R remain unclear. Therefore, to identify MPTP susceptibility to H_2_S preconditioning, we evaluated the CRC of mitochondria isolated from the liver after 24 h of reperfusion. As shown in Figure 5, a single preconditioning dose of 25 μmol/kg NaHS significantly improved the ability of mitochondria to tolerate calcium induction, which strongly improved the CRC, compared with the I/R group. Because MPTP opening is an important factor in determining whether I/R-induced cell death occurs during reperfusion, our findings suggest that H_2_S may protect hepatocytes from I/R injury by inhibiting MPTP opening.

### H_2_S suppresses cytochrome c release and caspase activation

**Figure 6:**
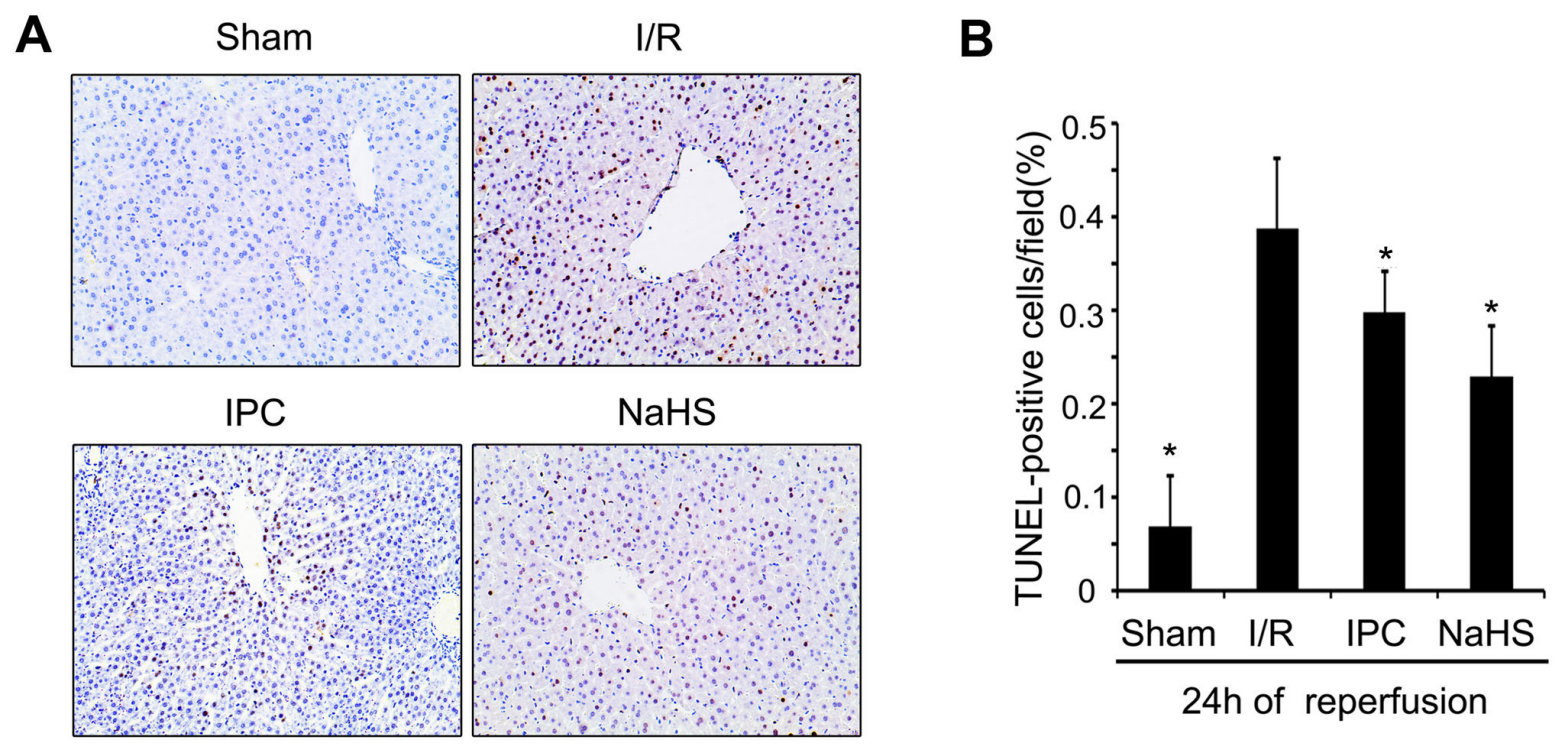
The effects of preconditioning with 25 μmol/L NaHS on hepatocyte apoptosis. Rats in the different groups were treated as described in Figure 1. (A) TUNEL staining of livers collected 24 h after reperfusion (100× magnification). (B) Bar graphs showing the percentages of apoptotic cells in tissue sections. At least six rats were included in each study group. The results are expressed as the mean ± SD. * *P* < 0.05 versus I/R.

**Figure 7:**
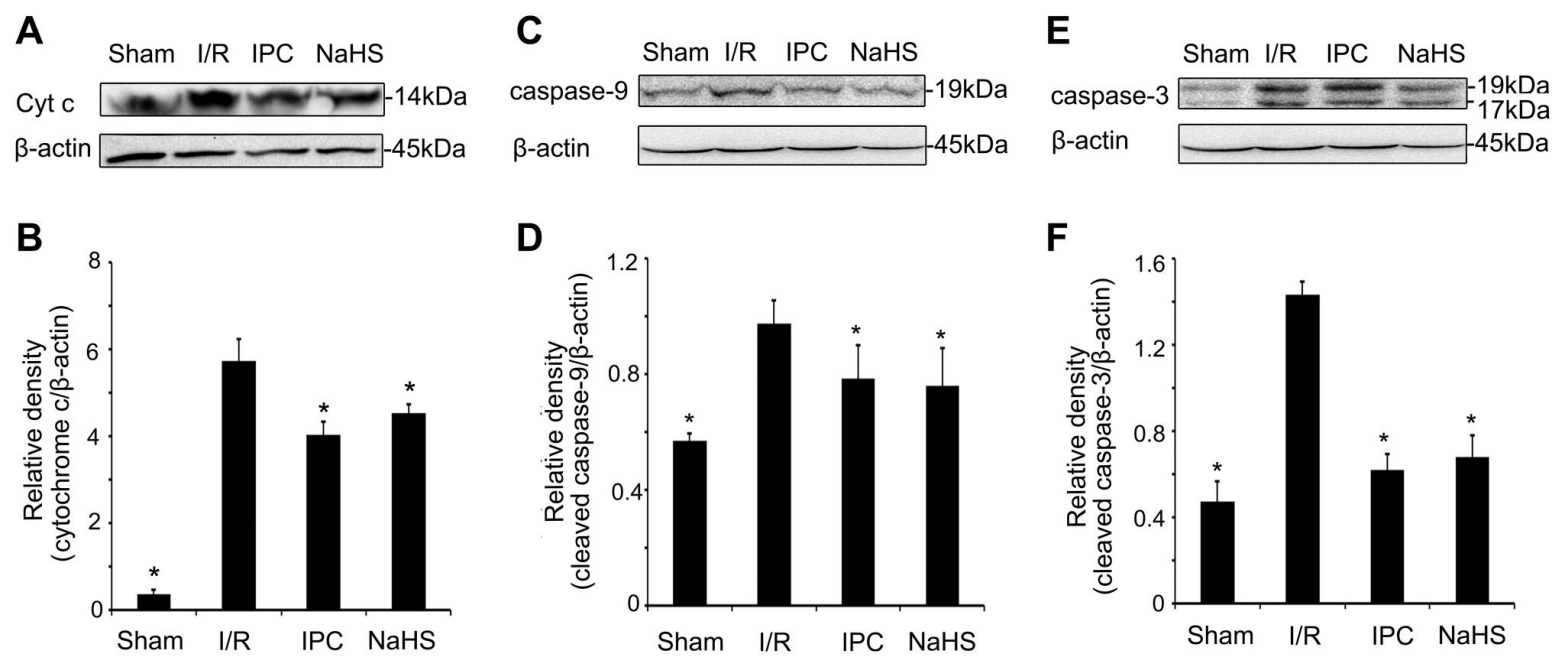
The effect of preconditioning with 25 μmol/L NaHS on cytochrome c release and caspase-9/3 activation. Rats in the different groups were treated as described in Figure 1. (A) A representative Western blot of cytoplasmic cytochrome c. (B) Relative levels of cytoplasmic cytochrome c. (C) A representative Western blot of cleaved caspase-9. (D) Relative levels of cleaved caspase-9. (E) A representative Western blot of cleaved caspase-3. (F) Relative levels of cleaved caspase-3. These experiments were performed in triplicate. The relative band densities are expressed as the mean ± SD. * *P* < 0.05 versus I/R.

MPTP opening causes mitochondrial-related cell apoptosis, which involves cytochrome c release and caspase activation [33]. Therefore, we next investigated the effect of H_2_S on apoptosis inhibition. TUNEL staining was performed to identify the effect of 25 μmol/kg NaHS on hepatocyte apoptosis. As showed in Figure 6A, a single preconditioning dose of 25 μmol/kg NaHS markedly reduced the TUNEL index (22.8% in NaHS rats versus 38.6% in I/R rats, *P* < 0.05). Furthermore, we investigated the effect of H_2_S on cytochrome c release and caspase-3/9 activation during hepatic I/R injury. Animals in the I/R group displayed increased levels of cytosolic cytochrome c expression compared with the Sham animals, while a dose of 25 μmol/kg NaHS administration prior to I/R insult greatly lowered the levels of cytochrome c released (Figure 7A). Cytochrome c release is associated with caspase family activation; therefore, we analyzed caspase-3 and caspase-9 cleavage with a western blot analysis. As expected, NaHS preconditioning markedly reduced the cleavage of caspase-9 (Figure 7B) and caspase-3 (Figure 7C). Taken together, these data suggest that H_2_S plays a role in preventing mitochondrial-related hepatocyte apoptosis by suppressing cytochrome c release and caspase activation during I/R injury.

### The effects of H_2_S on Akt-GSK-3β signaling

**Figure 8:**
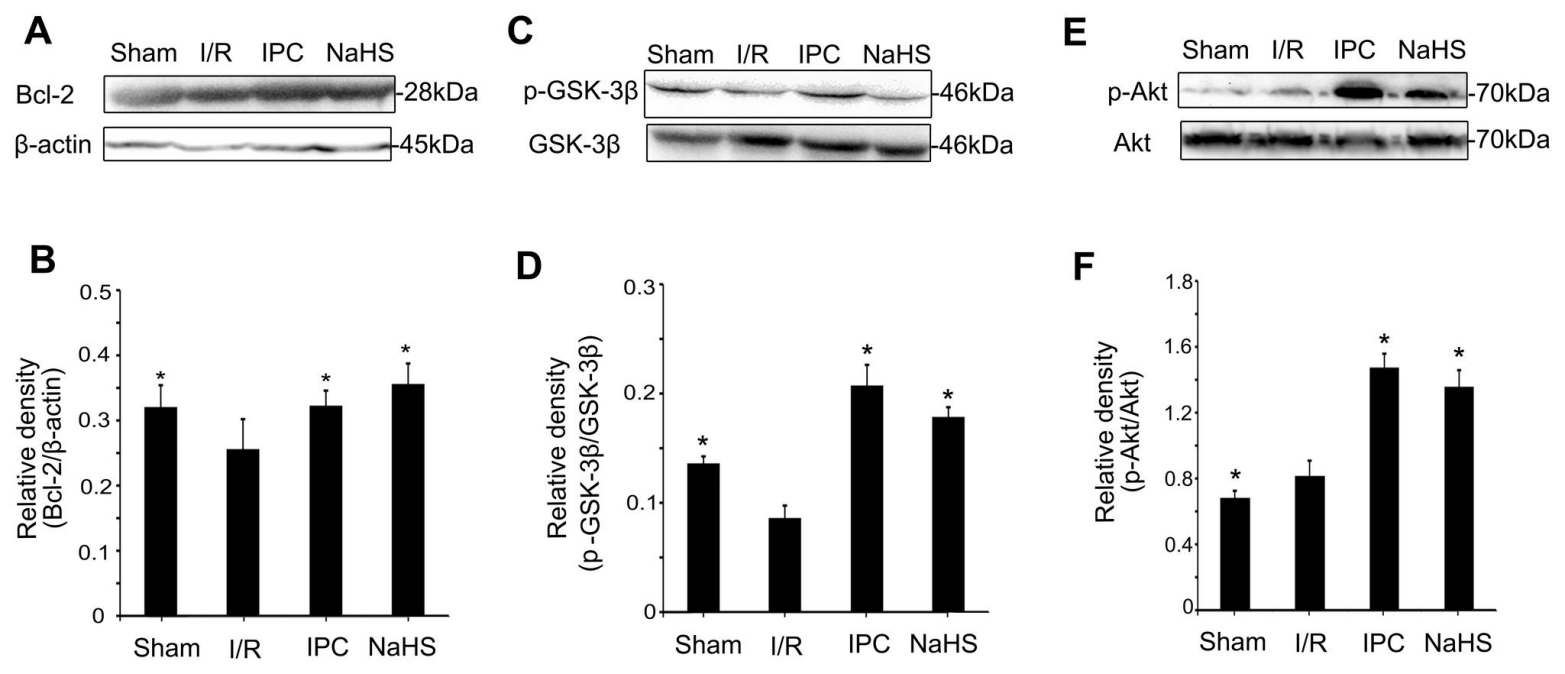
The effect of preconditioning with 25 μmol/L NaHS on the levels of Bcl-2, p-GSK-3β, and p-Akt. (A) A representative Western blot of Bcl-2. (B) Relative levels of Bcl-2. (C) A representative Western blot of p-GSK-3β and total GSK-3β. (D) Relative levels of p-GSK-3β and total GSK-3β. (E) A representative Western blot of p-Akt and total Akt. (F) Relative levels of p-Akt and total Akt. These experiments were performed in triplicate. The relative densities are expressed as the mean ± SD. * *P* < 0.05 versus I/R.

PI3K-Akt signaling and reperfusion injury salvage kinase (RISK) signaling are known to regulate the MPTP [10]. Akt has been shown to regulate members of the Bcl-2 family, which is composed of protective proteins involved in the mitochondrial apoptotic pathway. Moreover, Akt regulates the phosphorylation of GSK-3β [32,34], a pivotal enzyme implicated in MPTP regulation. Thus, we assessed the effect of preconditioning with 25 μmol/kg NaHS on Akt signaling in the liver after 24 h of reperfusion. As expected, NaHS preconditioning increased Bcl-2 (Figure 8A), p-ser9-GSK3β (Figure 8B) and p-Akt expression (Figure 8C), which indicates that NaHS preconditioning reduced MPTP opening by activating the PI3K-Akt-GSK3β signaling pathway.

## Discussion

IPC has been shown to alleviate hepatic I/R injury through the activation of protective signaling pathways and can be applied in clinical practice [26]. However, it may cause greater blood loss during the reperfusion period and a prolonged surgery course, which restricts its applications [35,36,37]. For this reason, an effective pharmacological approach for ischemia preconditioning is urgently needed. Recently, H_2_S has shown therapeutic potential in protecting against I/R injury.

Recently, a study that exposed mice to various concentrations of H_2_S demonstrated that with sub-toxic concentrations, there is a linear relationship between the concentration of inhaled H_2_S and an organ protection effect. The study also revealed that a high concentration of H_2_S has definite toxicity [13]. Therefore, no more than 100 ppm H_2_S gas was administered to the mice [23,38,39]. It is more accurate to administer NaHS by intravenous injection for animals such as rats and pigs. Sodha et al. found that intravenous administration of sodium sulfide (100 mg/kg bolus + 1 mg/kg/h continuous infusion) 10 min prior to the onset of reperfusion was cardioprotective during porcine cardiac I/R injury [40]. Intravenous administration of 0.2 or 0.4 μmol/kg H_2_S significantly decreased the apoplexy index, neurological symptom scoring, and infarcted areas of the brain in a dose-dependent manner in a rat model of cerebral I/R injury [41]. Yen et al. found that administration of 30 μmol/kg NaHS reduced infarct size and prevented cardiomyocyte apoptosis in a rat model of myocardial I/R injury [42]. In the present study, we evaluated the potential role of H_2_S in a model of 70% warm hepatic I/R. Our results showed that pretreatment with 12.5, 25, or 50 μmol/kg NaHS decreased ALT and AST levels in the plasma and that the higher two doses significantly decreased the Suzuki’s scores for the tissues (Figures 3 and 4). However, 33.3% of rats in the 50 μmol/kg NaHS group presented with dyspnea and died during the surgical procedure, which is likely caused by H_2_S-related lung injury, as Francis et al. previously reported [43]. Therefore, we decided to use a sub-toxic dose of NaHS (25 μmol/kg), which is similar to that used by Yen et al. [42], to further investigate the mechanisms of H_2_S on hepatic I/R.

Systemic hemodynamic alteration and organ blood supply may contribute to I/R injury. Previous research on a porcine model of cardiac I/R injury showed that intravenous administration of sulfide improved the noradrenaline responsiveness during reperfusion after aortic occlusion, implying that H_2_S may stabilize the hemodynamics in large-animal models [31]. However, there is no direct evidence that H_2_S has an effect on systemic dynamics. Our study confirmed that intravenous injection of 25 μmol/kg NaHS had no effect on systemic hemodynamics at various time points in a rat model of 70% warm hepatic I/R, which is widely used in studies focused on hepatic I/R [25,28,44,45,46,47]. Given that the hepatic portal system was not completely blocked (with the blood supply maintained in the right lobe and the caudate lobe), the blood returns from the postcava to the right atrium unaffected. Thus, this model causes few interruptions of the systemic dynamics and has a low mortality rate. Additionally, the ischemia phase lasted for only 60 min, which would have a comparably smaller impact to the long term ischemia insult, such as 90 or 120 min, on the systemic dynamics and microenvironment of the animal. Concordant results were found in a similar protocol (where the ischemia phase lasted for 30 min) [48]. This evidence implies that the protective effects of NaHS are not achieved by influencing the systemic dynamics. Thus, it likely works through different underlying mechanisms.

There are several molecular processes that are targeted by H_2_S to mediate injury protection [49]: (1) cell signaling, which plays various roles in anti-inflammatory and anti-apoptotic processes; (2) ion channels, specifically, activation of the K_ATP_ channel and inhibition of Ca^2+^ channels; (3) metabolism; and (4) protein modifications. The effects of these molecular targets provide evidence that H_2_S potentially mediates mitochondrial protection and thus prevents I/R injury. Although previous studies have shown that H_2_S preconditioning can up-regulate Bcl-2 expression in hepatocytes during I/R [22,23], the detailed mechanisms underlying H_2_S-mediated mitochondrial protection remain unclear.

Our data revealed that administration of a single dose of NaHS (25 μmol/kg) 5 min before ischemia significantly increased the H_2_S concentration in the plasma (Figure 2). Furthermore, similar to IPC, H_2_S pretreatment further protected rats against I/R-induced hepatic injury, as shown by the decreased serum levels of ALT and AST (Figure 3) and the maintenance of the normal morphological structure of liver cells (Figure 4). In addition, our results suggested that H_2_S preconditioning inhibited MPTP opening by improving the CRC (Figure 5) and reduced cell apoptosis (Figure 6) by inhibiting cytochrome c release and caspase-3 and caspase-9 activation during reperfusion (Figure 7). These findings provided strong evidence that, similar to IPC, H_2_S preconditioning preserves mitochondrial function and reduces mitochondria-mediated hepatocyte apoptosis.

Akt is an initiator of the downstream pathways that inhibit apoptosis. It phosphorylates Bad and ultimately inhibits cytochrome c release through blocking the channel formed by Bcl-2-associated X protein (Bax) in the mitochondrial membrane [50]. Moreover, Akt can phosphorylate GSK3β to prevent MPTP opening. Therefore, we examined the Akt-GSK-3β signaling pathway to elucidate how H_2_S modulates MPTP opening and mitochondrial function. We found that NaHS preconditioning significantly increased Bcl-2 and p-Akt levels (Figure 8A and Figure 8E). Members of the Bcl-2 family can regulate MPTP opening, and Bcl-2 can prevent MPTP depolarization [51,52]. Furthermore, our data indicate that NaHS preconditioning significantly enhanced Akt phosphorylation and GSK-3β phosphorylation at Ser9 (Figure 8B and Figure 8E). Previous studies demonstrated that GSK-3β phosphorylation at Ser9 leads to interactions with MPTP regulators and inhibits MPTP opening during reperfusion [3]. The present study demonstrates that H_2_S can increase Bcl-2 protein levels, inhibit MPTP opening, decrease activation of the cytochrome c-caspase-3/9 apoptosis pathway, reduce cell apoptosis and protect hepatic cells from I/R injury via activating Akt-GSK-3β signaling.

I/R-induced hepatocyte injury is a complicated process, and many aspects of damage are related to mitochondria. Therefore, the experiments presented here only addressed some major mechanistic pathways relevant to this process. Further research is required to explore additional mechanisms that may be involved.

## Conclusion

In conclusion, our data demonstrate a novel function for H_2_S whereby it inhibits MPTP opening and protects hepatic cells from I/R-induced injury. This discovery suggests that H_2_S could be a useful agent to preserve liver function in surgical settings, such as liver transplantation or tumor resections.
